# Learning History with Location-Based Applications: An Architecture for Points of Interest in Multiple Layers †

**DOI:** 10.3390/s21010129

**Published:** 2020-12-28

**Authors:** Samuli Laato, Sampsa Rauti, Antti Laato, Teemu H. Laine, Erkki Sutinen, Erno Lehtinen

**Affiliations:** 1 Department of Computing, University of Turku, 20540 Turku, Finland; sadala@utu.fi (S.L.); sjprau@utu.fi (S.R.); erkki.sutinen@utu.fi (E.S.); erno.lehtinen@utu.fi (E.L.); 2 Department of Teacher Education, University of Turku, 20540 Turku, Finland; alaato@abo.fi; 3 Department of Theology, Åbo Akademi University, 20540 Turku, Finland; 4 Department of Digital Media, Ajou University, Suwon 16499, Korea; 5 Department of Education, Vytautas Magnus University, 44248 Kaunas, Lithuania

**Keywords:** location-based applications, pervasive games, education, history, edge computing, crowdsourcing, point of interest

## Abstract

Location-based applications (LBAs) capture the user’s physical location via satellite navigation sensors and integrate it as part of the digital application. Because of this connection, the real-world environment needs to be accounted for in LBA design. In this work, we focused on creating a database of geographically distributed points of interest (PoIs) that is optimal for learning local history. First, we conducted a requirements elicitation study at three outdoor archaeological sites and identified issues in existing solutions. Second, we designed a multi-layered prototype solution. Third, we evaluated the solution with nine experts who had prior experience with LBAs or similar systems. We incorporated their feedback to our design to iteratively improve it. As a whole, our work contributes to the LBA design literature by proposing a solution that is optimized for the learning of local history.

## 1. Introduction

Today, satellite navigation sensors are ubiquitously embedded in smartphones. Together with internet connectivity, this has enabled location-based applications (LBAs) to become popular and widely used [1]. These applications link the user’s geographical location to the digital world. Examples of popular LBAs include navigation software (e.g., HERE drive, Google Maps, Navman) and games, such as Pokémon GO and Orna. Because LBAs provide a digital 2D representation of real world geography, they can be transformed into augmented reality (AR) applications by including fictional things to the map interface [2,3]. For example, the location-based game Pokémon GO, interprets real buildings as part of the game world but additionally superimposes fictional creatures in the map interface [4].

Some LBAs, particularly games, have the benefit of naturally motivating mild exercise in the form of walking, cycling, or otherwise moving around [5]. This characteristic makes them interesting from an educational standpoint, as they are a welcome change to the often necessary and currently widely used forms of learning where learners need to sit still. Recent work has highlighted LBAs’ potential to teach about the environment the users are in, for example, in the form of showing information about the surrounding buildings or by directing LBA users to local points of interest (PoIs) [6]. A study by Huizenga et al. demonstrated that LBAs can be harnessed to teach history, which increased students’ motivation to learn [7]. In this type of design, where players learn about their surroundings by walking in historical locations, it is important that the virtual PoIs are of high fidelity, contain reliable information, and are correctly placed [8]. While such sets of PoIs can be created for individual places or cities [7], the global maintenance of such a PoI database requires thousands of hours worth of human resources [9].

While scholarly work has demonstrated LBAs, and, more specifically, location-based AR games, to be useful in history education (e.g., Reference [7,10]), the main issue with these solutions is that they only work at specific locations, not globally, and a large amount of work would be required to extend them to world wide coverage. To solve this problem of labor, the successful existing solutions have relied on crowdsourcing [9], that is, sourcing the manual labor to volunteers around the world. OpenStreetMap (OSM) is an example of a crowdsourced [11] global map [12], whereas some other maps (e.g., Google Maps) are a mixture of manual work from a company and input of its users and business owners. With regard to location-based games, the current market leader in global PoI creation and management is the location-based technology focused company Niantic. Their crowdsourced PoI database is primarily created for the purpose of being a platform for games [13,14] and is almost entirely user-generated, with some part of the review process and the entirety of the technical solution being taken care of by Niantic [9]. Niantic provides players with interfaces for both submitting and reviewing PoIs, and also enables players to vote for suggested edits to the PoIs. From the above listed four challenges for creating a global PoI database for LBA-based education, Niantic’s solution resolves fairly well the first two issues. However, the database and its criteria have been shown to discriminate against minority inhabited areas [15] and to lack quality PoIs in some areas, such as archaeological sites [8]. Furthermore, the database contains PoIs only in a single layer, lacking historical depth, and it struggles to motivate players to meaningfully contribute to the solution. Based on extant literature on the topic, these challenges may be summarized as follows:

1. Thousands of hours of manual labor is needed to provide PoIs and related metadata without automation [14]. Crowdsourcing and gamification are promising [9,16], but they can also escalate into cartographic vandalism [17].
2. Technical support and solutions for creation, as well as maintenance, are needed [9].
3. Criteria need to be decided. What are the optimal criteria for accepting PoI candidates? Will this create an uneven divide of PoIs between areas? How to solve the problems arising from an uneven distribution [13,14,15]?
4. How to differentiate between labor force expertise? How to best utilize the expertise of, for example, archaeologists for outdoor archaeological site PoIs [8,18]?

Accordingly, technical problem solving and innovation are needed to create a global PoI database that is historically relevant and which LBAs can use to support the learning of local history. Research on the topic is still at its infancy, and work is needed to establish the design requirements of such a database. Work is also needed in optimizing existing solutions. To address these research problems, we first conducted a design elicitation study [19] in the context of three archaeological sites in the Levant: *Gezer*, *Hazor*, and *Megiddo*. Next, we designed a PoI database creation and maintenance scheme that can be used to fulfil the identified design requirements. We evaluated the proposed solution by comparing it to currently available solutions using the use case view [20]. Third, we contacted experts (N = 9) who had prior experience with LBAs and PoI solutions to evaluate our proposal. With these three approaches, this work makes the following contributions:

1. Establishes the design requirements for a virtual geographical PoI database that has the primary aim of scaffolding the implicit learning of local history.
2. Proposes a technical solution for the creation and maintenance of such database.
3. Provides a formative evaluation of the system through collected expert feedback.

## 2. Research Design and Methodology

In the design requirements elicitation part of this work, we focus on three archaeological sites located in the country of Israel: *Tel Hazor*, *Tel Megiddo*, and *Tel Gezer*. All three places appear in the First Book of Kings, Chapter 9, verse 15 and have been of interest to archaeologists with several excavations taken place [21,22]. These sites have ruins of ancient structures which have been discovered in multiple strata, such as those dated to Late Bronze Age and Iron Age [23]. All three sites are outdoor locations and are currently open for visitors. *Tel Hazor* and *Tel Megiddo* have been declared World Heritage Sites, meaning their conservation has been recognized internationally to be of great importance. We focus on how these locations appear in OSM and the Niantic PoI database. As a consequence of this analysis, we derive a set of design requirements for a LBA database focused on implicit teaching of local history.

The PoI database used in Niantic’s *Ingress* game was chosen for analysis as the database is global [14], the virtual PoIs match real-world locations [13], and PoIs are visible for all in the Ingress Intel Map [24]. Besides *Ingress*, the same PoIs are largely used also in other games, such as *Pokémon GO* and *Harry Potter: Wizards Unite* [13]. Furthermore, applications based on this database have been found to increase players place attachment [6], thus providing preliminary evidence towards LBA’s potential for enhancing visitors experience at cultural sites. The three archaeological sites were looked up in the *Ingress Intel Map* in October 2019 and later again in October 2020 together with OSM data. All found PoIs, their title, and location were recorded, and, based on these characteristics, they were mapped to corresponding real-world objects. If the PoI title was in another language than English, such as Arabic or Hebrew, it was translated to English. As a comparison and tool for analysis, information of the sites was obtained from the *Israel Nature and Parks Authority* [21] website, as well as major publications on the archaeological findings and their scholarly interpretations. The virtual PoIs found in *Ingress* were analyzed by looking at (1) what kind of a PoI is it? (ruin, sign, model), (2) from which time period or stratum is it from?, and (3) which archaeological interpretation does it represent? The virtual PoIs were then compared to the actual visible structures.

In the second part, following the design elicitation, an iterative design science approach [25] was used to design a multi-layered PoI database that has temporal layers of geographically distributed PoIs. Then, using knowledge from previous research, the requirements elicitation and expert feedback, we improved the solution. This is also connected to our third part, where we harness expert feedback to evaluate the system. To this end, we created a video presentation (8 min 27 s) to explain our solution and uploaded it to YouTube as an unlisted video. We embedded the video into a survey created with Webropol (Helsinki, Finland). We included the following questions:

1. Can this kind of a solution be used to teach local history? Why/why not?
2. Do you believe the solution can be an improvement upon existing solutions? Why/why not?
3. What challenges do you see in implementing this kind of a system in practice?
4. For what purposes can this solution be used in addition to location-based games?
5. Do you have any improvement suggestions to the proposed solution?

The participants were recruited among active contributors to the Niantic crowdsourced PoI system, as well as University personnel who have worked with LBA research and development in South Western Finland. All participants were asked for a permission to participate in the research and promised that their responses would only be used anonymously in the reporting. In addition to the expert feedback, we evaluated our system through using the case view of Kruchten [20,26] and analyzing how the system could be optimized via the use of edge computing.

## 3. Design Requirements Elicitation

### 3.1. Case Study: Archaeological Locations

Annually millions travel to see archaeological sites of cultural, historical or religious significance. These sites are typically outdoors and are prepared for visitors after archaeological excavations are completed [27]. Pottery and other smaller artifacts found on the excavation site or nearby may also be put on display, as well as models or reconstructions of predicted historical structures. To supplement the artifacts visible on site, signs, guidebooks, audio-guides may be offered to visitors. AR applications and games can also be added including gamification, scientific interpretations and additional info about the location [28,29].

#### 3.1.1. Tel Hazor

Lead by Yigal Yadin, major archaeological excavations took place at Tel Hazor in the 1950s, which revealed bronze and iron age structures and evidence of both Canaanite and, then later, Israelite settlement [30,31,32,33]. The site has been of interest to biblical scholars, archaeologists, and historians [31] and has been studied together with several other similar ancient ruins in the region [33,34]. The largest individual remaining structure in Tel Hazor is an underground water system, which was discovered by Yadin’s later 1968–1969 expeditions and has been dated to the Iron-age [35]. Similar water systems have been found in several cities on top of mountains from the same time period [35]. Another major structure is a *“Salomonic city gate”*, dating of which has been discussed by scholars to be either from the time of Salomon (10th century BCE) or the Omrid dynasty (9th Century BCE) [34]. In addition, other structures, mostly interpreted as housing, remain on site [36], including a typical 8th century BC Israelite four-room house [37,38].

**Figure 1:**
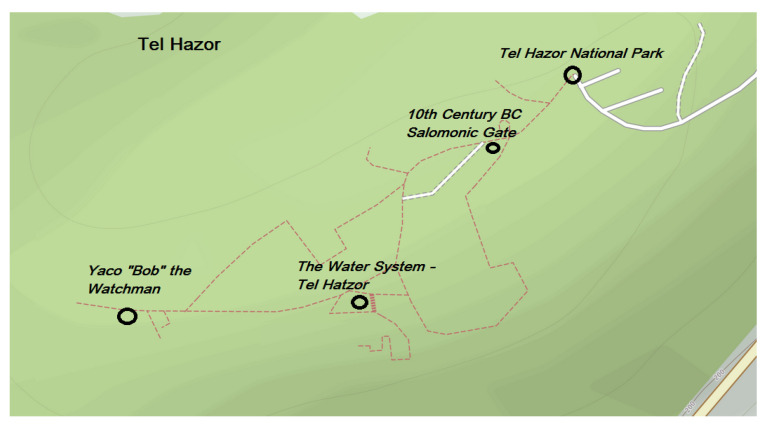
A view of Niantic points of interest (PoIs) in Tel Hazor. PoIs are observed in the Ingress Intel Map and depicted on top of OpenStreetMap (OSM). Image constructed by the authors on 30 October 2020.

Figure 1 shows the locations and names of all virtual PoIs (4) of Tel Hazor currently in the Niantic PoI database. Two of the PoIs, *10 Century BC Salomonic Gate* and *The Water System- Tel Hazor*, point to ancient historical artifacts. *Yaco ‘Bob’The Watchman* shows a modern art piece depicting an ancient Israelite Guard, and the final PoI *Tel Hazor-National Park* is a reference to the entire site. It is evident these PoIs only lightly touch the historical depths of this location, as multiple structures, such as the Israel four-room house, are not included as virtual PoIs, and the information of the existing PoIs is limited. For example, with regard to the *10 Century BC Salomonic Gate*, only the interpretation of Yadin and Ben-Tor is shown, even though Finkelstein dates the structures to the period of the Omrides dynasty, as seen in Table 1. With regard to OSM, it displays 10 PoIs in Tel Hazor, a more detailed view compared to the Niantic solution.

#### 3.1.2. Tel Megiddo

Megiddo is a world heritage site located on a mountain in the middle of the Jezreel plains and has been featured in pop culture due to its association to the Armageddon, apocalypse, and the end of the world [39]. Among the most massive constructs, Tel Megiddo site contains a deep water system [40] from the Iron Age period, similar to those found in *Hazor* and *Gezer* [35], as well as the ruins of a great temple dated to the early Bronze Age (3000 BCE) [41]. Tel Megiddo has arguably the most detailed data in all of Levant for the period from Late Bronze (3000 BCE) to Iron Ages (750BCE), thus having unparalleled historical value [23].

**Figure 2:**
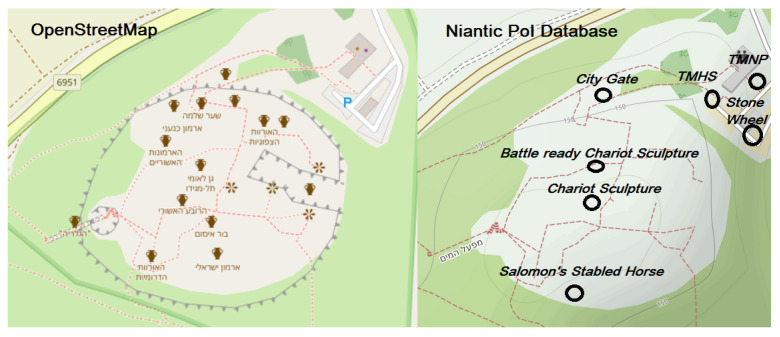
Depicting the PoIs in Tel Megiddo by OSM, as well as the Niantic PoI database. Image constructed by the authors on 30 October 2020.

Figure 2 shows a side-by-side comparison of PoIs in OSM (screenshot taken by the authors) and Niantic virtual PoIs (7) displayed on top of the same OSM background. From the comparison, we see that the PoIs in OSM are much more detailed but still do not depict he entire archaeological richness of the location. The Niantic PoIs are much more generic and contain only a single historical location: the city gate. Three of the Niantic PoIs are signs: *Tel Megiddo*, *Tel Megiddo World Heritage Site*, and *Tel Megiddo National Park*. Then, there are three scultupres: *Battle Ready Chariot Sculpture*, *Chariot Sculpture*, and *Salomon’s Stabled Horse*. Unlike in *Hazor*, the PoIs in *Megiddo* do not offer direct references to ancient structures, except for the city gate. For example, the water system is not a PoI, and neither is the ruin of the Bronze Age (3000 BCE) Canaanite temple [41]. Furthermore, the naming of the PoIs and their descriptions do not depict that there is an ongoing scholarly debate on the dating of the strata VB and VA-IVB (see Table 2).

#### 3.1.3. Tel Gezer

Ancient Gezer was an important strategic area due to its geographical location guarding Via Maris, Valley of Aijalon and the trunk road leading to Jerusalem [42]. Excavations began at the site in 1902, lead by Robert Alexander Stewart Macalister, and lasted seven years [43]. More excavations have since taken place, such as Alan Rowe’s six-week campaign in 1934 and The Hebrew Union College Excavations in 1964–1966 [43]. Structures from multiple strata dating to Late Bronze Age and Iron Age have been discovered from the location [33,42,44], including a Salomonic four-entryway city gate, similar to which is also found in Tel Hazor and Tel Megiddo [42]. However, the Gezer gate is a bit different in it being based on a square plan instead of a rectangular one [34]. A Canaanite water tunnel has also been found in the ruins, along with Masseba stone structure and many other smaller structures.

Seven PoIs were discovered at Tel Gezer in the Niantic PoI database. These PoIs were named in Hebrew and are roughly translated by the authors as (1) Sheikh Aljazarli’s Tomb, (2) Area of Worship: Masseba Site, (3) Salomon Gate, (4) Canaanite Gate, (5) Water System, (6) Map of the vicinity of Tel Gezer, and (7) Gezer Calendar. Compared to the other two observed locations, *Tel Gezer* has the largest quantity of virtual PoIs representing ancient structures in the Niantic database, exceeding the four PoIs shown in OSM. Yet, for example, the debate regarding the chronology of the structures is not visible. Similarly to virtual PoIs in *Tel Hazor*, Finkelstein’s Iron Age low chronology [45,46] is dismissed (see Table 3). In OSM, Tel Gezer shows four PoIs. Thus, here, it shows less PoIs than the Niantic solution, differentiating the location from Tel Hazor and Tel Megiddo.

### 3.2. Design Requirements

Based on the analysis of the three archaeological sites, we propose four design considerations, as follows.

#### 3.2.1. Ensuring the Quality and Fidelity of the Virtual PoIs

Virtual PoIs should cover the key real-life PoIs on the site to support learning of local history. *Ingress* currently allows PoIs to have a short description and photos, in addition to their name and location, while OSM displays no additional information. When aiming for historical accuracy, creating high fidelity historical PoIs requires expert knowledge. In the same way, contributions to the description of PoIs and the relations between them would require further elaboration by experts of history education. Here, automatic solutions can fall short unless they make use of already existing information [14]. The alternative is to use crowdsourcing, or a mixture of automatic procedures and crowdsourcing.

#### 3.2.2. Support for Visualizing Multiple Layers of PoIs

When looking at the three case archaeological sites of *Hazor*, *Megiddo*, and *Gezer*, a common challenge is that there exists competing views among scholars with regard to interpretations of the excavated structures’ dating and original purpose. One of the questions with regard to the observed three locations has been whether or not the great fortification systems with gates mentioned in *The First Book of Kings 9:15* can really be dated to the reign of Solomon (i.e., to time of united monarchy) or whether they should be dated a little bit later to the Omride dynasty in the kingdom of Israel (e.g., Reference [47,48]). This discussion highlights how scholars have dated stratigraphic layers differently at archaeological sites and, consequently, interpreted the origin and purpose of discovered structures in various ways.

Because scholars may disagree on interpretations of archaeological evidence, it is important to accurately present evidence of all cases for visitors. However, the existing solutions enable only the visualization of a single layer of PoIs [2,13]. This is also problematic from the perspective of visualizing various historical era. In the paramount reality, only one reconstruction can be presented at a time [3], but AR technology and LBAs can solve this issue as the reconstructions are digital and can be switched at will. For example, a broken ancient wall, which depending on interpretation was either an arc or just a wall, can be displayed as both.

#### 3.2.3. Information on Lost Objects and Structures

Several excavations, such as those that have taken place in Tel Hazor [27,31,49,50,51], have revealed structures from multiple time periods across many strata. Furthermore, when archaeologists dig deeper to reveal older structures, they are forced to remove strata on top. As a result of this process, many excavation sites are left with structures from multiple strata to display. AR gives the possibility of viewing the same place through various lenses, each depicting information from a certain era [52]. This also connects to the previous theme in that the PoI database should provide support for multiple layers of PoIs. It is equally important that the PoI database contains information that no longer exists in the paramount reality. For example, at archaeological sites, when strata are removed to dig to older layers, the lost information could be captured and displayed in AR instead as one form of conservation of knowledge. Furthermore, the system could enable showing two related objects from different eras in AR, enabling side-by-side comparison of how the place has evolved during the years.

#### 3.2.4. Design of Crowdsourcing to Expand the Solution into Global Scale

Based on observing the Niantic Wayferer system that is used to peer-review PoI submissions for their database, we notice a few key issues. The challenges of the system lie in that (1) editing of PoI locations is a long and unpredictable process; (2) portal candidates submitted too close to existing ones are not included in the visible PoI database; (3) players are motivated to create PoIs close to them and demotivated for accepting PoIs close to their opponents; (4) the peer-reviewers of PoI candidates are selected among players and, in most cases, are not experts in evaluating the descriptions; and (5) the PoI criteria are designed and communicated to players in such a way that historically valuable locations can easily be rejected. These are but examples of potential issues that may arise in expanding a PoI database to become global via using crowdsourcing [16]. However, open source software projects, Wikipedia, OSM, and other crowdsourcing success stories have proven that it is a viable strategy if implemented correctly [11]. Yet, even Wikipedia and others can encounter problems, such as a few individuals coming to dominate a vast amount of content [53] and vandalism [17].

## 4. Preliminary Solution: A Multi-Layered PoI Database Creation Scheme

### 4.1. Visualizing Historical Layers, Information, and Interpretations

**Figure 3:**
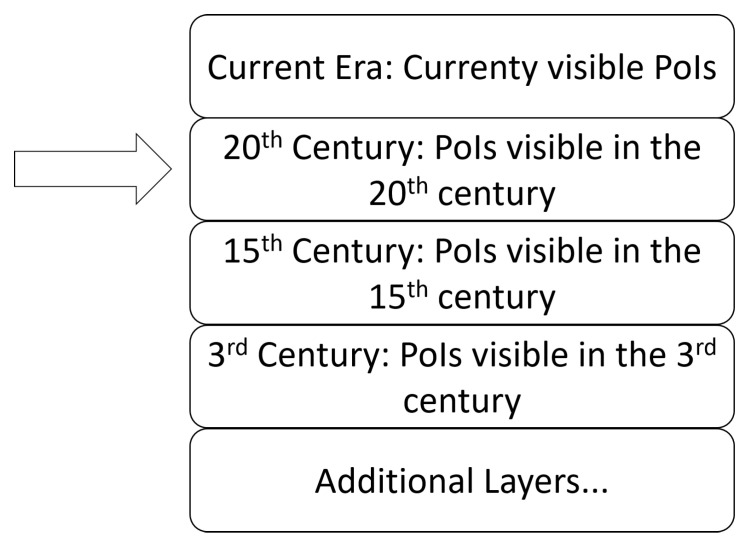
With a multi-layered PoI database that has information of historical PoIs, location-based application (LBA) users can re-imagine what their surroundings looked like centuries ago.

The first thing to address, that is particularly relevant at archaeological sites, is the problem of visualizing structures that no longer exist. For example, several historical buildings have been destroyed and at archaeological sites excavations to deeper strata require the destruction of what is on top. However, having currently visible and destroyed structures displayed on a map interface for LBA users can be confusing. Furthermore, this would make it more difficult for users to visualize what their environment looked like at a given era. For this reason, we propose that a PoI database for teaching local history should be divided into layers. The topmost layer would represent structures that are currently visible in the world. In addition to this layer, historical layers would be included. LBA users could choose whether they want to see PoIs of the current era, or a from previous era. This is displayed in Figure 3.

In these layers, PoIs could be placed on the locations where structures historically resided in that time period, similarly to how they are displayed in Figure 1. Thus, switching between the historical layers would display different PoIs, although it is perfectly possible that some buildings existed in multiple eras and are thus displayed on multiple layers. The layers that are accessible would be determined based on the location. For example, a rich historical city could have way more layers than a newly founded town. LBAs using the PoI database could use it in various ways; for example, games could require players to reach certain goals in order to unlock further layers.

Having multiple layers of PoIs enables visitors to view what the place would look like in the eyes of Finkelstein [45] and in the eyes of Mazar [54]. But, it also enables visitors to see how it looked like in the middle Bronze Age and in the late Iron age. An additional advantage of this multi-layer approach is that it is future proof in that new layers and interpretations can be added, and PoIs never become obsolete once correctly added. A possible disadvantage is that, when there are more layers, managing and presenting them in a clear way becomes increasingly complex.

### 4.2. PoI Criteria

One of the most important aspects of these types of solutions is the criteria based on which the PoI are submitted and accepted in the database. Several sets of criteria have been proposed by previous works (e.g., Reference [13,14]) which rank objects based on their importance, so that a large cathedral is a preferred PoI over a tree. For example, Niantic uses man-made-structure as a prerequisite for PoIs to be accepted to their database. For a PoI database aiming for both temporal and geographical depths, we see such limitations to be unnecessary. The fields where historical battles took place, as well as mountain tops where ancient settlements resided, should be valid PoIs as they have historical value. Another aspect regarding the PoI criteria is the distance between PoIs. Depending on the LBA that uses the PoI database, too short a distance between PoIs can be limiting. To this end, LBAs could have the opportunity to only display a set of PoIs from the database, but, for the database itself, no limit to the distance between PoIs is needed. In order to teach history to LBA users, we propose the following acceptance criteria for PoIs.

1. The PoI needs to have historical significance.
2. It needs to represent a historical structure or event that took place in the geographical location that it is placed in.
3. The PoI needs to have a descriptive name and should not contain fabricated information, and, when possible, the information should be verified by experts.
4. The rough dates of the event or structure the PoI represents can be included.

### 4.3. Populating the PoI Database: Automation, Crowdsourcing, and Expert Knowledge

For a global database that not only has the current layer but several historical layers, the amount of labor required to accurately map the entire world and its history is immense. However, the task is particularly suitable for crowdsourcing [11,16]. Using an existing crowdsourced map, such as OSM, as a backbone is useful for supporting crowdsourcers in placing the PoIs to correct geographical locations [14]. LBA users can also be harnessed as an edge computing resource for crowdsourcing, which we name here as *“edge sourcing”*. End users are harnessed as on site experts to document and compute new additions to the PoI database.

Among tactics for motivating people to participate in crowdsourcing of the solution, gamification [9,13] has been widely used in previous work. Harari explains that blank spots on world maps hugely motivate people to travel and fill in those spots [55]. Giving credit to those who contribute and having a blank map are, therefore, promising solutions for participant motivation. In order for the review process to work, all participants need to be motivated to be on the same side [16]. A cross-team conflict, such as that in the Niantic’s games Ingress and Pokémon GO, can cause players of opposing teams to develop negative feelings towards each other [56], which can increase sabotage of the PoI database or underlying map systems [17]. Furthermore, religious and ideological conflicts of the real world can interfere in objective analysis of locations [57]. As a remedy and to assure the quality of the PoI database, universities and other accredited institutions can be provided with a fast track for submitting and removing PoIs, enabling the institutions which have the purpose or harboring knowledge to more efficiently contribute to the database.

## 5. Expert Evaluation

Altogether, nine participants replied to the survey. All participants agreed to give permission to use their responses anonymously in this work. The participants were all experienced LBA users, with experience about the Niantic PoI database solution and OSM. Participants were aged between 25–60 and both male and female.

### 5.1. Advantages

Participants saw potential in this solution for teaching local history. The most prominent given reason was that, as the system would be tied to historical locations, learning history in some way while using our solution would be inevitable. For example, one participant commented as follows: *“In a game [using this database] many crucial locations would be a part of local history: famous places, birthplaces, meaningful infrastructure locations, artworks etc.”*. Another expert commented: *“This solution is better [than the Niantic’s solution or OSM], because here the PoIs are specifically tied to the theme of learning history.”*

### 5.2. Potential Issues

The experts highlighted the complexity and difficulty of maintenance as the potential key issues in our proposed system. One participant explained: *“The biggest challenge lies in how to create the simplest working basic structure for the system. Another challenge relates to recruiting experts for the proposed expert verification and maintenance within the system.”* Another participant raised an issue regarding the PoI criteria and the need to be more specific there: *“Where do you draw the line what is culturally or historically significant and deserves to be a PoI?”*. Finally, issues related to the maintenance of the system were proposed, i.e.,: *"Perhaps the biggest challenge would be how to control, display and use the collected PoIs in applications."*

### 5.3. Improvement Suggestions

All participants responded with some improvement suggestions. The following four were estimated by the authors as welcome additions to the system: (1) *“There should be a color coding to the PoIs in such a database. Not all PoIs are the same, and there should be tags defining what kind of PoI it is.”*; (2) *“Just create the database based on openly available information (e.g., Wikipedia), get coordinates from there and place them in the database. Half of the job done.”*; (3) *“Gamification can help motivate people to contribute (see the Niantic solution). So as an improvement suggestion, you could think whether such a database is created first and then used, or created as it is used via crowdsourcing.”*; and (4) *“Clarification is needed on who can input data to the database. What kind of a registration is required? Who controls it?”*.

## 6. Use Case View of the Final Proposed Solution

**Figure 4:**
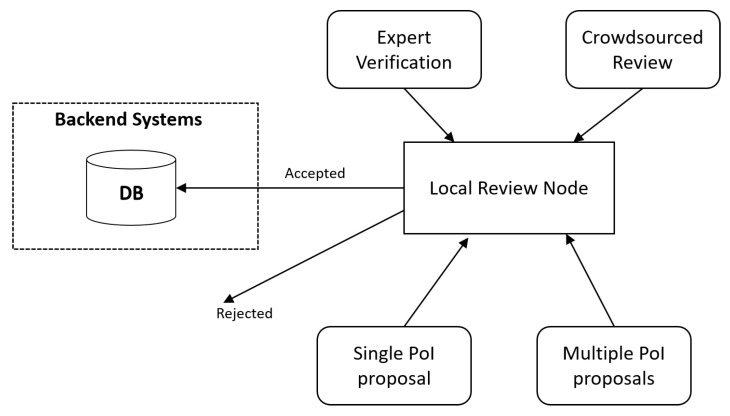
A visualization of the PoI submission process in our system that is optimized with the use of edge computing.

Based on the expert evaluation, we made final adjustments to the proposed solution. There are three use cases [26] of our PoI database scheme that we discuss in this section. First is the basic suggestion and review loop in PoI creation and maintenance. This is displayed in Figure 4. LBA users can submit either individual PoIs or multiple PoIs for review. The reviews can be provided either by experts or crowdsourced to other LBA users. In case the reviews are favorable, the PoI suggestion will be moved to the global backend systems for further processing. The local review node operates as an edge computing resource that is able to significantly reduce the computational load and traffic to the main backend systems. In addition, data transmission is faster between users and the local node in comparison to if users communicated directly with the global backend systems.

**Figure 5:**
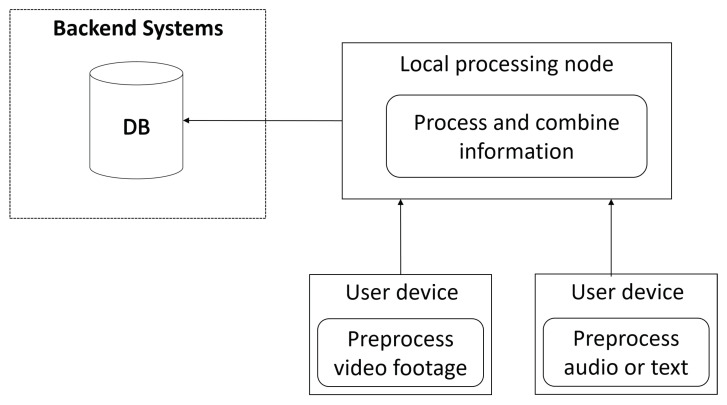
An outline of how edge computing is used in two stages when submitting metadata and supplementary information to the PoI database.

The second use case relates to adding supplementary information to existing PoIs. Once the temporal and geographical dimensions of a PoI are established, LBA users can see them on a map. Upon traveling to these PoIs, users have the option to add data about the PoI and submit it onward for processing and validation. In Figure 5, we visualize two users’ devices, one sending video data and the other sending audio or text files. Audio and video files can be processed locally by first converting them to file formats that take little space and then by extracting metadata from them using machine learning or other types of techniques. This pre-processed data can then be sent over to the local node for further processing before finally being added to the global database. By utilizing edge computing in this manner, the system load can be split among crowdsourcers, which also saves bandwidth as less data needs to be sent over the internet.

The third use case is the use of the PoI database. When a LBA requests information related to a PoI, a local edge node can return this information directly from a cache or local database without a need to make a request to a backend database. This decreases latency and reduces global traffic. Updating PoIs to global database does not need to be fast because reviewing the PoIs manually will take time anyway and propagating PoI changes quickly is not important in history-themed LBAs. Therefore, delivering PoI data to the backend from the edge and syncing the backend database with the local edge nodes can be done when the system is not under heavy load.

## 7. Discussion

### 7.1. Comparison with Previous Solutions

There are two main types of implementations to which our system can be compared to. The first is previous LBAs that have been designed for history education. Viinikkala et al. created a location-aware AR adventure game taking place in a cathedral, where users could relive stories from the past [10]. The solution contains high fidelity AR but is local and can only be played in a specific location. Huizenga et al. [7], likewise, created a game for learning history; however, their solution Frequency 1550 was city-wide. These solutions were limited in both the temporal and geographical dimensions. Neither was global nor provided a platform for spontaneous learning of local history across eras. Our solution is superior in this regard, but it loses in ability to utilize more context specific technologies, such as physical sensors placed on specific locations or game design that relies on total control of a physical space.

The other solution type that our system needs to be compared to are existing map services, such as OSM [17] and, in particular, the crowdsourced PoI database created by Niantic [9]. Our solution has the important advantage of this work in that it has multiple layers of PoIs, enabling experiencing complete historical eras at once. One important criticism towards this proposition is that getting historically relevant high fidelity PoIs with crowdsourcing would be increasingly difficult in this case as populating layers of history with accurate PoIs requires expert input. As a remedy, we included a fast track for experts, such as accredited universities, to contribute to the system as displayed in Figure 4.

### 7.2. Challenges in the Real World Implementation

Additional discussion is needed on the technical implementation. This relates to ethical, legal and privacy issues that are connected to the crowdsourcing solution and data collection [58]. For example, as people are invited to contribute images to the PoI database, verification is needed to make sure the images are not copyrighted and are in fact unique. Some images taken by players may contain other people, so blurring their faces would be needed to avoid privacy infringement. As people are possibly taking video footage from real world objects and edge computing is used to process this data, questions regarding the consumption of network bandwidth, the mobile device memory, and computing power arise. Here, we need to balance the technical implementation with what is convenient to the user and to the system in a way that is ethical.

In this work, we discussed mainly outdoor locations, but it is worth considering that valuable historical information and content exist also within buildings, such as old cathedrals, churches, museums, castles, and caves. To this end, techniques involving indoor localization (e.g., Reference [59,60]) need to be utilized. To expand this solution to cover indoor historical locations, further engineering work is needed. Here, in addition to standard global positioning system (GPS) sensors, more precise sensors (e.g. bluetooth, li-fi, lidar, gyroscope, and other sensors) could be used to determine the mobile device orientation and location accurately within indoors. One additional technical problem that needs to be addressed is the synchronization of the local data node to the global database.

It also needs to be discussed how to enable experts to contribute to the solution. Without additional funding, experts at universities may not have the resources or interest to contribute effort to such a database. Here, one resource that could be utilized are university students. Bergström [61] describes how the LBA Pokémon GO could be integrated as an observational study activity at universities. Along the same lines, students of history could be tasked to contribute to such a PoI database as part of their studies as field work. This could benefit students as they learn, as well as teachers and the University as their reputation increases, and, of course, LBA users as they receive higher quality PoIs.

### 7.3. Pedagogical and Practical Considerations

The way our solution teaches local history is partly based on implicit learning, i.e., automatic learning while using LBAs. As users travel to PoIs while using LBAs, they absorb information and can get prompted to learn more [62]. As such, this PoI database is an optimal backbone for LBAs as it introduces implicit learning benefits with little to no cost on usability. However, there is very little evidence of the effects of implicit learning on deeper conceptual learning in history [63]. Accordingly, the hypothesized learning benefits need to be rigorously evaluated in future studies. A second benefit is that users get to learn local history while walking around, introducing physical exertion to history education. A third benefit from the educational standpoint is that potential for gamifying learning, which can boost students’ motivation. Students’ can even use LBAs while socially interacting, enabling them to communicate with one another while travelling to historical PoIs.

This kind of a system for LBAs could also have negative consequences. Having PoIs at world cultural heritage sites might attract unwanted attention. For example, the database could be used as a backbone for applications whose users have no regard for the site they are walking at. This could, in worst cases, cause damage to the place. Furthermore, not all places are suitable for LBAs at all. As an example, the Auschwitz concentration camp, a museum for the Jewish holocaust, has completely forbidden the playing of location-based games on their grounds [64].

### 7.4. Limitations and Future Work

In this work, we presented a design elicitation, as well as a design of a multi-layered geographical and historical PoI database and its evaluation. In the design elicitation, we chose a particular geographical and historical context, archaeological places in the Levant, for analysis. We focused on the Niantic PoI database and OSM at these sites and compared how well they manage to take into account the historical structures in these locations. As such, the design elicitation was operationalized in a quite specific context. To counter this limitation, the design elicitation could be carried out in other context and also methods invoking knowledge from LBA users could be used. There are also limitations in the system design. At this stage, the design is preliminary and needs to be implemented for rigorous testing. This is an obvious limitation. With regards to the expert evaluation, we were limited by the number of participants and our ability to describe the solution to participants.

One of the most important topics for future work remains the empirical validation and testing of the proposed solution. Because it heavily relies on crowdsourcing, a simple small-scale proof of concept would be insufficient to adequately test its feasibility. In addition, several ethical considerations arise from this work. First, crowdsourcing harnesses the crowds to do work but without employee benefits or protection. Second, using individuals’ phones as edge computing resources imposes strain on their hardware. Users might not expect or realize that their phones are being used for pre-processing of data. Thus, users should be able to choose to opt out of pre-processing data, in which case raw data would need to be sent over the internet, which, then again, could increase network bandwidth consumption.

### 7.5. Conclusions

The proposed PoI database solution has potential in teaching local history, but, as such, it cannot replace traditional history education where global history is taught. Still, it can transform the teaching of local history to be more engaging and interactive and also enhance visitors’ experience at cultural outdoor sites with rich history, such as the archaeological sites which we observed in the design elicitation. The multi-layered geographical database gives more depth to the currently used temporally uni-layered solutions in popular LBAs, such as Pokémon GO. As pervasive computing, smart cities, smart environments, and digitization of our daily lives moves onward, scientists and technology designers need to constantly not only create new solutions, but to seek ways to make use of the available infrastructure as well. Here while our solution was designed with the purpose of implicit local history learning, it can have other benefits as well such as bringing LBA users together to specific PoIs facilitating social interaction and motivating people to walk to PoIs scaffolding physical activity.

## Figures and Tables

**Table 1:** Comparison of chronological and historical explanations of ruins and artifacts discovered in strata X and IX in Tel Hazor.

	Yadin and Ben-Tor	
**Stratum**	**Dating**	**Historical Setting**
X	10th Century BCE	Salomon
IX	Late 10th, Early 9th	Israelite
	**Finkelstein**	
**Stratum**	**Dating**	**Historical Setting**
X	Early 9th Century BCE	Israel: Omrides
IX	First half of 9th Century BCE	Israel: Omrides

**Table 2:** Comparison of chronological and historical explanations of ruins and artifacts discovered in strata VB and VA-IVB in Tel Megiddo.

	Yadin and Mazar	
**Stratum**	**Dating**	**Historical Setting**
VB	10th Century BCE	United Monarchy
VA-IVB	Late 10th Century	Salomon
	**Finkelstein**	
**Stratum**	**Dating**	**Historical Setting**
VB	About 900 BCE	Early Israelite Monarchy
VA-IVB	First half of 9th Century BCE	Israel: Omrides

**Table 3:** Comparison of chronological and historical explanations of ruins and artifacts discovered in strata IX and VIII in Tel Gezer.

	Dever	
**Stratum**	**Dating**	**Historical Setting**
IX	10th Century BCE	Salomon
VIII	Late 10th, early 9th	Israelite
	**Finkelstein**	
**Stratum**	**Dating**	**Historical Setting**
IX	10th Century BCE	No evidence for united monarchy
VIII	First half of 9th Century BCE	Israel: Omrides

## Data Availability

The data presented in this study are available on request from the corresponding author.

## Abbreviations

The following abbreviations are used in this manuscript:

PoI	Point of Interest
LBA	Location-based application
AR	Augmented reality
OSM	OpenStreetMaps
GPS	Global positioning system
